# Perventricular double-device closure of wide-spaced multi-hole perimembranous ventricular septal defect

**DOI:** 10.1186/s13019-017-0585-5

**Published:** 2017-04-17

**Authors:** Fei Liang, Li Hongxin, Hai-Zhou Zhang, Guo Wenbin, Cheng-Wei Zou, Zeeshan Farhaj

**Affiliations:** 1 Department of Cardiovascular Surgery, Provincial Hospital Affiliated to Shandong University, No. 324 Jingwu Road, Jinan, 250021 China; 2 Ultrasound Department, Provincial Hospital Affiliated to Shandong University, Jinan, China

**Keywords:** Perimembranous ventricular septal defect, Device closure, Echocardiography, Perventricular

## Abstract

**Background:**

Device closure of a wide-spaced multi-hole PmVSD is difficult to succeed in percutaneous approach. This study is to evaluate the feasibility, safety and efficacy of perventricular device closure of wide-spaced multi-hole PmVSD using a double-device implanting technique.

**Methods:**

Sixteen patients with wide-spaced multi-hole PmVSD underwent perventricular closure with two devices through an inferior median sternotomy approach under transesophageal echocardiographic guidance. The largest hole and its adjacent small holes were occluded with an optimal-sized device. The far-away residual hole was occluded with the other device using a probe-assisted delivery system. All patients were followed up for a period of 1 to 4 years to determine the residual shunt, atrioventricular block and the adjacent valvular function.

**Results:**

The number of the holes of the PmVSD was 2 to 4. The maximum distance between the holes was 5.0 to 10.0 mm (median, 6.4 mm). The diameter of the largest hole was 2.5 to 7.0 mm (median, 3.6 mm). The success rate of double-device closure was 100%. Immediate residual shunts were found in 6 patients (38%), and incomplete right bundle branch block at discharge occurred in 3 cases (19%). Both complications decreased to 6% at 1-year follow-up. Neither of them had a severe device-related complication.

**Conclusions:**

Perventricular closure of a wide-spaced multi-hole PmVSD using a double-device implanting technique is feasible, safe, and efficacious. In multi-hole PmVSDs with the distance between the holes of more than 5 mm, double-device implantation may achieve a complete occlusion.

## Background

Percutaneous device closure of perimembranous ventricular septal defect (PmVSD) has been shown to be safe and efficacious. However, device closure of a wide-spaced multi-hole PmVSD is difficult to succeed in percutaneous approach. Patients with a wide-spaced multi-hole PmVSD were usually converted to surgical repair under cardiopulmonary bypass.

In recent years, perventricular single device closure of PmVSDs has been developed and applied clinically with good results [1–4]. However, no reports have been published on device closure of a wide-spaced multi-hole PmVSD with two devices. The purpose of this study is to evaluate the feasibility, safety and efficacy of perventricular closure of wide-spaced multi-hole PmVSD using a double-device implanting technique and to comment on the midterm follow-up results of this technique.

## Methods

### Patients’ clinical details

Between October 2011 and July 2015, 16 patients underwent perventricular double-device closure of wide-spaced multi-hole PmVSDs through an inferior median sternotomy approach in our hospital. Baseline noninvasive data were obtained by physical examinations, electrocardiography, transthoracic echocardiography (TTE), and chest radiography.

Eight patients had a mild or moderate tricuspid regurgitation; 5 patients had a mild or moderate mitral regurgitation; 2 patients had a mild aortic regurgitation; 4 patients were treated after failure in percutaneous closure. Indications for multi-hole PmVSD closure were the same as those used for surgical closure, which included hemodynamically significant left to right shunts, left ventricular chamber enlargement, and (or) mild to moderate pulmonary hypertension.

The inclusion criteria for double-device closure of multi-hole PmVSDs included: 1) Age of 12 months or older; 2) The largest hole diameter of PmVSD of more than 1.5 mm; 3) The residual hole diameter of PmVSD of more than 1.5 mm; 4) The distance between the holes of PmVSD of more than 5 mm; and 5) Left to right shunt.

Exclusion criteria included: 1) Patients with the sum of the diameter of all the holes of less than 3 mm; 2) Contraindications to antiplatelet therapy; and 3) Those coexisting with other cardiac anomalies.

The enrolled patients or their guardians hoped to close the defect, eliminate the heart murmur, and have a cosmetic procedure. Once a patient met the enrollment criteria, he or she or the guardian was fully informed of the available treatment options. Informed consent was obtained from each patient or his or her parents. The study was approved by the ethics committee of our institution and performed in compliance with the institutional guidelines and those of the American Physiological Society. The baseline characteristic data of 16 patients are shown in Table 1.

**Table 1:** Baseline clinical characteristic data and procedural data

No	Sex	Age (y)	Weight (kg)	LDVSD (mm)	Number of holes	Size of holes (mm)	MD (mm)	1^st^ device (mm)	2^nd^ device (mm)	ICMT (min)	Procedure time (min)
1	M	39	80	19	4	7.0,2.0,2.5,1.5	7.2	9,sc	5,wc	68	135
2	M	1	9	8	2	2.6,2.0	7.0	4,sc	4,wc	45	75
3	M	1	12	9	2	2.7,2.5	5.3	4,sc	4,sc	50	88
4	M	3	18	12	2	6.0,2.5	9.0	8,sc	4,sc	47	115
5	M	3	16	14	4	5.6,2.5,1.5,1.5	10.0	8,sc	5,wc	48	100
6	M	20	56	18	2	3.2,2.5	6.5	6,sc	4,sc	90	160
7	F	3	14	12	2	2.5,2.5	5.5	4,sc	4,wc	45	84
8	M	19	64	15	2	3.5,2.5	6.0	6,sc	4,sc	65	108
9	M	3	13	11	2	2.8,2.2	6.1	4,sc	4,sc	53	100
10	M	6	19	13	3	3.2,2.0,1.5	5.0	7,wc	4,sc	40	99
11	F	1	7	10	3	2.5,2.0,1.5	5.3	5,sc	4,sc	45	120
12	F	1	12	9	3	2.5,2.0,1.5	5.5	5,wc	4,sc	33	80
13	F	8	25	17	2	3.8,2.5	6.5	6,sc	4,sc	60	105
14	F	5	28	10	2	3.0,2.3	5.2	5,sc	4,sc	50	98
15	F	13	54	15	2	3.5,2.5	5.8	6,sc	4,sc	62	108
16	M	4	19	9	2	3.0,2.5	5.3	5,sc	4,sc	38	70

*LDVSD* Left-sided diameter of ventricular septal defect, *MD* maximum distance between the holes, *sc* concentric short-edged occluder, *wc* concentric wide-edged occluder, *ICMT* intracardiac manipulation time

### Device and delivery system

The devices used in this study are modified ventricular septal occluders (Starway Medical Technology, Inc, Beijing, China), based on the Amplatzer septal occluder. There are two types of devices used in this study: concentric short-edged or wide-edged occluder with the left disc 2 mm or 4 mm larger than the connecting waist. The right disc is 2 mm larger than the waist and the waist is 3 mm long in both types of devices.

The device selection is determined according to the type, size, and the subaortic rim of the PmVSD as follows:(1) In the small aneurysmal type of PmVSDs, two short-edged occluders are selected with the size 1 to 2 mm larger than the diameter of each hole. (3) In the large aneurysmal type of PmVSDs, a wide- or short-edged occluder is selected to occlude the largest hole with the size 2 mm larger than the hole. The residual hole is occluded with a short-edged occluder with the size 1 to 2 mm larger than the hole.

**Fig. 1:**
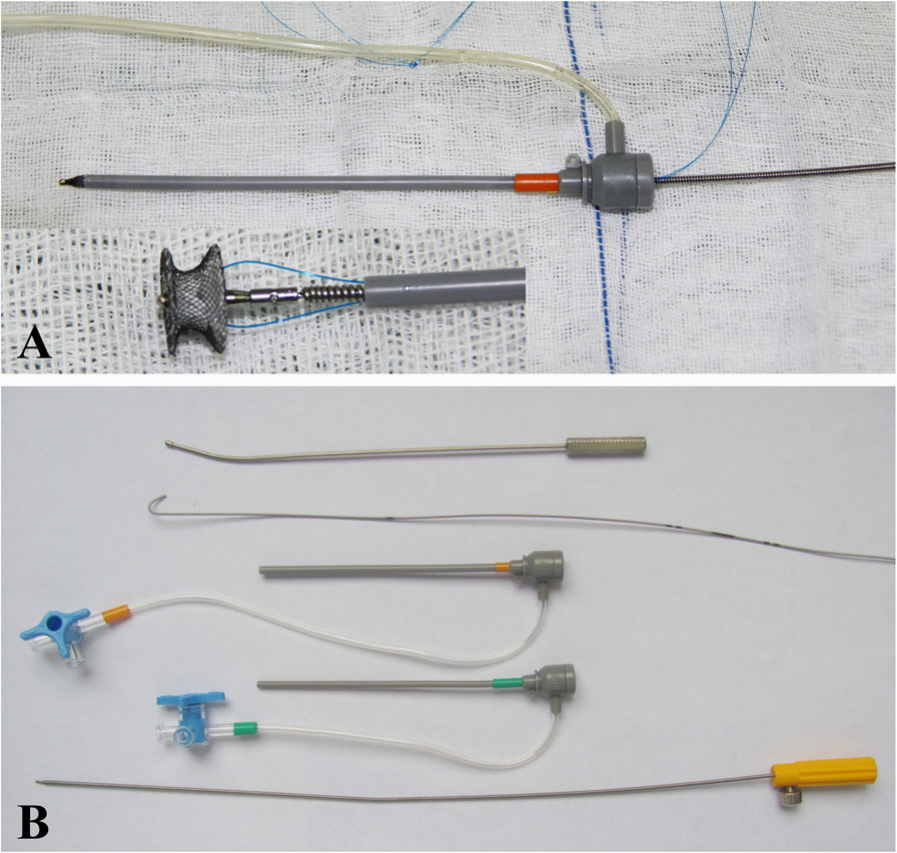
**a** The direct delivery system consists of a short delivery sheath and a delivery cable. (magnification of inset 2.5 ×) **b** The probe-assisted delivery system consists of a hollow probe, a flexible guidewire, a delivery sheath, a loading sheath and a delivery cable

Two types of delivery systems are available in this study [5, 6]. (1) The probe-assisted delivery system (PADS) consists of a hollow probe, a flexible guidewire, a delivery sheath, a loading sheath, and a delivery cable. The probe is malleable, about 20 cm in length, with an olive tip and a channel throughout the whole length. The delivery or loading sheath is about 10 cm to 15 cm long, with a side arm for removal of air and a size ranging from 5 F to 8 F (Fig. 1). (2) The direct delivery system (DDS) consists only of a short delivery sheath with a side arm for removal of air and a delivery cable (Fig. 1).

### Transesophageal echocardiography (TEE)

A Philips IE33 echocardiography instrument (Philips Healthcare, Best, Netherlands) with a 2.0- to 7.0-MHz frequency conversion probe was used. After general anesthesia and intubation, patients were placed in a supine position. A complete TEE evaluation was performed to determine the shape, size, and location of the defect. All patients were screened in the left ventricular long-axis view and five-chamber view to measure the diameter of the left-sided and each hole of the defect. The subaortic rim was also measured. The integrity of the adjacent valves was assessed simultaneously.

### Procedure

All patients were placed in a supine position and draped for exposure of the entire chest. A 2.5 to 4.0 cm lower mini-sternotomy incision was made. Exposure was optimized with a miniretractor. The pericardium was opened and suspended to expose the right ventricle. The puncture site was chosen by pressing the right ventricular free wall with a peanut sponge under TEE monitoring. The site in which the pressed shadow protruded towards the middle point between the largest and the remote holes of the defect was determined and two parallel pursestring sutures of 5/0 or 4/0 polypropylene (Ethicon, Somerville, NJ) were placed at this site. The largest hole was occluded using a DDS. The selected device, connected with a device stay suture [5, 6], was retracted into the delivery sheath directly. After anticoagulation with heparin (1.0 mg/kg), a right ventricular puncture was made within the pursestring sutures. The delivery sheath loaded with the device was inserted into the right ventricle and advanced through the largest hole of the PmVSD into the left ventricle. Then the device was deployed following the previous reports [3, 4, 6].

**Fig. 2:**
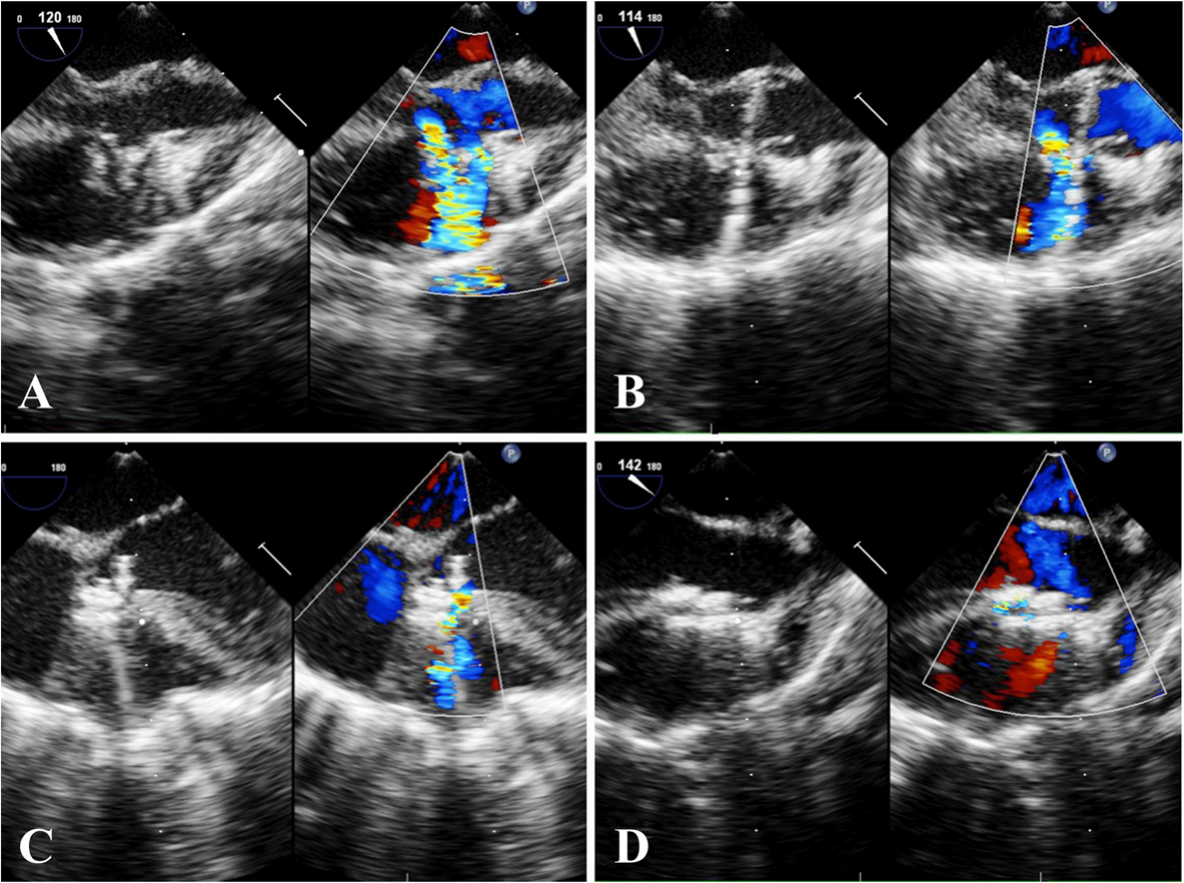
**a** The PmVSD had two holes, and the maximum distance between the holes was more than 5 mm; **b** The largest hole was occluded with the direct delivery system; **c** The residual hole was occluded with the probe-assisted delivery system; **d** The double devices were positioned properly, and there was no residual shunt

After failing complete occlusion with single wide-edged occluder at the first attempt, the double-device implanting procedure was determined. The largest and residual holes were reassessed (Fig. 2a) ,and the implanting devices were reselected. Sometimes the implanted wide-edged occluder was replaced with a same sized, short-edged occluder to provide enough space for the second device. After the first device was positioned and released (Fig. 2b), the device stay suture was not removed until the second device was positioned properly.

The residual hole was occluded using a PADS (Fig. 2c). From the same puncture site, the bendable hollow probe was inserted into the right ventricle and adjusted to aim at or cross the residual hole. A flexible guidewire was advanced through the channel of the probe into the left ventricle. The subsequent steps were as previously reported [3, 4, 6]. When TEE evaluation showed there were no significant residual shunt and no significant aortic or tricuspid valve regurgitation, the second device, connected with a device stay suture beforehand, was released. After proper positioning of both devices was confirmed (Fig. 2d), their device stay sutures were removed. The pericardium was re-approximated, and a central venous catheter used as a drainage tube was placed in the pericardium. The incision was closed in layers.

The VSD size, implanted device size, intracardiac manipulation time, procedure time, the residual shunt, left ventricular end-diastolic diameter, and postoperative hospital stay were recorded after procedure and during the follow-up period.

### Patient follow-up

**Fig. 3:**
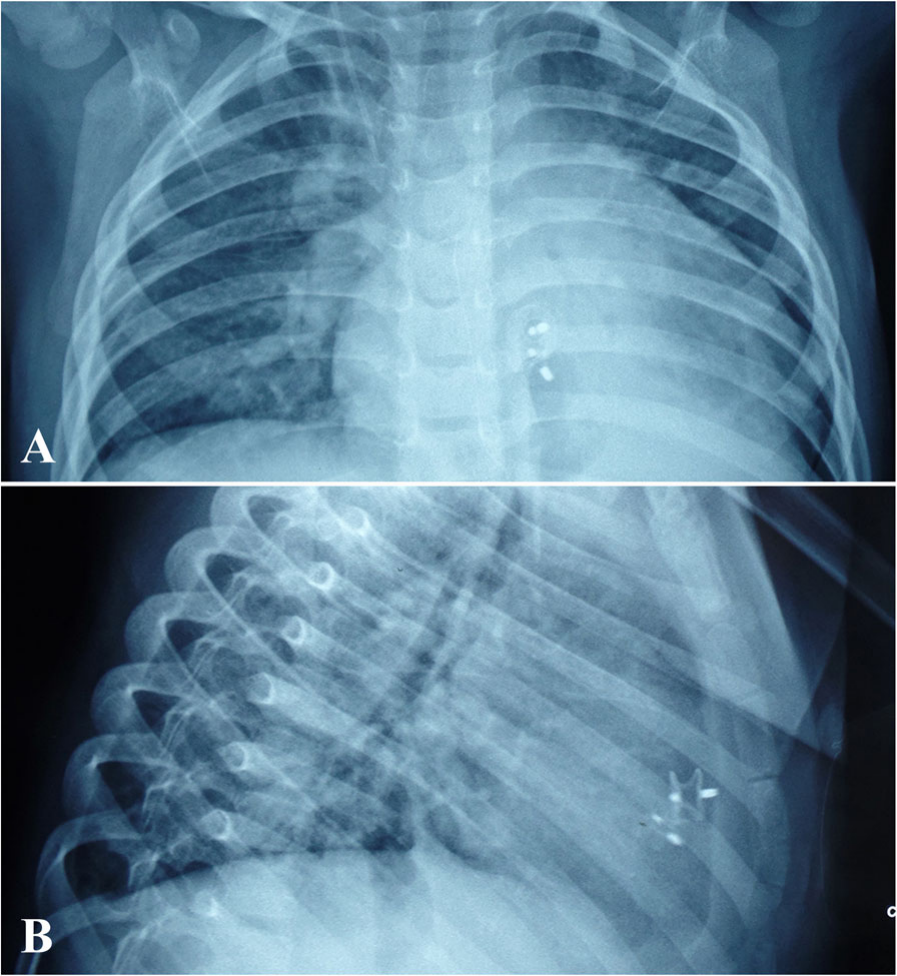
The chest radiography showed the arrangement and a good configuration of the two devices. **a** AP view; **b** Lateral view

Prophylactic antibiotics were started before the procedure and continued for 2 days. Most patients were discharged 3 to 5 days after the procedure and maintained on aspirin (3–4 mg/kg/d) for the duration of 3 months. The follow-up protocol included assessments by electrocardiography, TTE and chest radiography (optional) (Fig. 3) at discharge, 1, 3, 6, 12 months and yearly after the procedure.

### Statistical analysis

Data were expressed as median (or mean ± standard deviation) and range. Intracardiac manipulation time was defined as the time: the right ventricle was punctured until the delivery sheath and cable were withdrawn from the right ventricle. Statistical comparisons of proportions were analyzed using a chi-square test (Stata10.0 software; StataCorp LP, College Station, TX). A probability value of less than 0.05 was defined as statistical significance.

## Results

### Intraoperative results

The success rate of double-device closure of multi-hole PmVSD was 100%. The complete closure rate at device release was 62.5%. The number of the holes of the PmVSDs was 2 to 4. The maximum distance between the holes was 5.0 to 10.0 mm (median, 6.4 mm). The diameter of the largest hole was 2.5 to 7.0 mm (median, 3.6 mm). The sum of all the holes’ diameter ranged from 4.6 to 13.0 mm. The intracardiac manipulation time was 53 ± 14 min, and the procedure time was 102 ± 25 min. The procedural data and outcome of the 16 patients are listed in Table 1.

### Postoperative and follow-up results

Postoperative tracheal intubation time was 2 to 4 h, and postoperative hospital stay was 4 to 6 days. The left ventricular end-diastolic diameters before and after the operation were: 4.4 ± 1.1 cm (range, 2.7 to 6.8 cm) vs 3.8 ± 0.9 cm (range, 2.6 to 5.8 cm) (*P* < 0.05). All patients were followed up for a period of 1 to 4 years with TTE and electrocardiography—16 patients for 1 year,11 for 2 years, 7 for 3 years, and 3 for 4 years. The follow-up rate was 100%. Immediate residual shunts were found in 6 patients (38%), and incomplete right bundle branch block (IRBBB) at discharge occurred in 3 patients (19%). Both complications decreased to 6% at 1-year follow-up. The preexisting mitral, tricuspid and aortic regurgitation remained unchanged, or showed a decrease in its severity during the follow-up period. Neither of them had a device dislocation, complete atrioventricular block and aortic or tricuspid valve impingement. The follow-up data are listed in Table 2.

**Table 2:** Postoperative and follow-up results

Variable	IADR	At discharge	1 Month	6 Months	12 Months	24 Months	36 Months	48 Months
Preexisting AR (%)	2/16(13)	1/16(6)	0/16(0)	0/16(0)	0/16(0)	0/11(0)	0/7(0)	0/3(0)
New AR (%)	0	0	0	0	0	0	0	0
Preexisting TR (%)	8/16(50)	7/16(44)	4/16(25)	1/16(6)	1/16(6)	1/11(9)	0/7(0)	0/3(0)
New TR (%)	0	0	0	0	0	0	0	0
Preexisting MR (%)	5/16(31)	2/16(13)	2/16(13)	1/16(6)	0/16(0)	0/11(0)	0/7(0)	0/3(0)
New MR (%)	0	0	0	0	0	0	0	0
New IRBBB (%)	–	3/16(19)	2/16(13)	1/16(6)	1/16(6)	0/11(0)	0/7(0)	0/3(0)
Mild residual shunt (%)	6/16(38)	5/16(31)	3/16(19)	1/16(6)	1/16(6)	0/11(0)	0/7(0)	0/3(0)

*AR* aortic regurgitation, *TR* tricuspid regurgitation, *MR* mitral regurgitation, *IRBBB* incomplete right bundle-branch block, *IADR* immediately after device release

## Discussion

### Advantages of the perventricular approach

Perventricular device closure of a muscular and PmVSD through a median sternotomy approach was firstly reported by Amin and colleagues in 1998 [7]. Since then, this technique has been increasingly developed and is becoming an effective alternative to surgery [3, 6, 8, 9]. Although peratrial or perventricular device closure of PmVSDs through a right or left parasternal approach has been reported [6, 10], the lower median sternotomy approach is still the preference due to its simplicity, reliability, and the perpendicular short entry route to the septum.

Percutaneous device closure of a concentrated multi-hole PmVSD using single device is safe and efficacious [11, 12]. However, it is infeasible to occlude a wide-spaced multi-hole PmVSD with two devices percutaneously. After the first device is positioned, it is difficult for the guidewire to cross the residual hole because most of the residual holes are partially covered by the first device. Especially in patients with a first implanted wide-edged occluder, the residual-defect crossing from left to right is impossible and may entrap the guidewire. Even the second arteriovenous guidewire loop were established, the second delivery sheath might impinge on the first device and cause its dislodgement when passing through the residual hole.

Compared with the percutaneous approach, the perventricular approach has many advantages, including: 1) the perpendicular short entry route, which permits easy defect crossing and proper device positioning; 2) No weight and no vascular access limitations; 3) excellent manipulability of a short, rigid, straight sheath or probe; 4) no need to establish an arteriovenous guidewire rail, which avoids chordal and trabecular entrapment; 5) simple process of recapture and redeployment of the device using the device stay suture; and 6) no exposure to radiation.

### Advantages of double-device implanting technique

In multi-hole PmVSDs with a distance between the holes of more than 5 mm, it’s impossible to close all the holes completely using single occluder because the left disc of the wide-edged occluder available is only 4 mm larger than the connecting waist. Traditionally, these patients had to choose surgical repair under cardiopulmonary bypass. The double-device implanting technique using DDS and PADS can achieve the goal of closing all the holes completely. The PADS and DDS have the advantages as follows:

- First, both of them are simple, which decrease the cost of the procedure.
- Second, the DDS simplify the device closure process. Because of the excellent manipulability, the DDS loaded with the device, generally permits direct entry into the large hole without using the probe, guidewire, and loader.
- Third, the PADS makes it easy to cross the residual hole from right to left. The hollow probe can approach the residual hole with very good echocardiographic visualization. It improves the possibility of crossing the defect and minimizes the risk of entrapping the guidewire by the first implanted device. In some patients, the jet of the residual hole is towards the right atrium or apex, the distal end of the probe can be made an angle so that it can point towards the jet to introduce the guidewire to cross the defect.
- Fourth, the risk of injuring the cardiac valves or other structures is decreased. The PADS and DDS enter the defect directly without passing through any cardiac valve, which avoid chordal and trabecular entrapment.
- Fifth, The device stay suture has improved the safety of this technique. It has the ability to retrieve a suboptimally placed device through a larger delivery sheath and prevents device dislocation and embolization after device release.

Compared with surgical repair, the double-device implanting technique has the advantages of: 1) less trauma, 2) cosmetic small incision, 3) no need of cardiopulmonary bypass; 4) short procedural time, Intensive Care Unit stay and hospital stay, and 5) no blood requirement.

### Device-related complications

1. Complete atrioventricular block
  Several studies have reported that the incidence of complete atrioventricular block is approximately 1% to 6.5% in the percutaneous approach [11, 12], and 0 to 3.3% in the perventricular approach [3, 4, 8, 9]. A complete atrioventricular block occurring immediately during the procedure may result directly from mechanical injury or compression caused by catheter or devices. Endomyocardial edema and chronic fibrosis are more likely to be responsible for the late-onset complete atrioventricular block. In this series, no patients presented with complete atrioventricular block, but IRBBB before discharge occurred in 3 cases (19%). Its incidence is slightly higher than that of single occluder (approximately 15%) [3, 6, 9, 13]. However, it showed a decrease during the follow-up period. At 1-year follow-up, only 1 case had IRBBB. We think that IRBBB is caused by the oppression of closure device and endomyocardial edema at early stages. It is very important to reduce the endomyocardial friction in operation and choose an optimal occluder as possible.
2. Impingement on the adjacent valves
  The ventricular septal occluder may affect the function of the aortic valve. During the procedure, if a new aortic regurgitation was found, the device would be retrieved and replaced with a small or eccentric occluder [10, 12–14]. In this study, no device-induced aortic regurgitation was found because the double devices were placed in the pouch of the PmVSD aneurysm far from the aortic valve. Compared with about 10-20% of new tricuspid regurgitation reported in the perventricular approach [3, 13, 14], no device-induced tricuspid regurgitation occurred in this technique. The reason might be that we chose two small devices to occlude the exit of the PmVSD instead of a large single occluder which may cover more area of the tricuspid valve and affects its function.
3. Residual shunt
  Immediate trivial or mild residual shunts were found in 6 patients (38%) in our study, which was higher than the incidence of about 15-25% reported in the literature [1–4, 12–14]. The reasons may include: 1) most of the residual shunts were inter-disk shunts; 2) the PmVSD may had 3 or more holes and two of them were occluded which resulted in smaller insignificant residual shunts from incompletely oppressed smaller holes; 3) the closely positioned double devices limit their stretch with each other in the early postoperative period, which causes inter-device residual shunt. It is generally believed that trivial or mild residual shunts detected immediately after device release usually disappear during the follow-up period. Endothelialization will cover the surface of the device, and neointima will form and fully close the inter-disk shunts. The self-expandable devices stretch completely and close the adjacent small holes and inter-device shunt. At 1-year follow-up, only 1 case had trivial residual shunt.
4. During the follow-up period of 1 to 4 years, no device-related thrombosis or embolism occurred. The perventricular double-device implantation did not increase the risk of thrombus embolization.

### Study limitations

This study is not a prospective randomized study. We just speculated its safety and advantages according to the previous reports, experience and the results in our study. This technique is just suitable for patients with wide-spaced multi-hole PmVSD. We had only 16 successful patients in this series and the follow-up period was only 1 to 4 years. Further studies are required to establish long-term results in a larger patient population.

## Conclusions

Our study has demonstrated that perventricular closure of a wide spaced multi-hole PmVSD using a double-device implanting technique is feasible, safe, and efficacious. It had the advantages of less trauma, quicker recovery time and better cosmetic results. In multi-hole PmVSD with the distance between the holes of more than 5 mm, double-device implantation may achieve complete occlusion.

## Abbreviations

DDS: The direct delivery system
IRBBB: Incomplete right bundle branch block
PADS: The probe-assisted delivery system
PmVSD: Perimembranous ventricular septal defect
TEE: Transesophageal echocardiography
TTE: Transthoracic echocardiography
